# Tumor-Activated Mesenchymal Stromal Cells Promote Osteosarcoma Stemness and Migratory Potential via IL-6 Secretion

**DOI:** 10.1371/journal.pone.0166500

**Published:** 2016-11-16

**Authors:** Margherita Cortini, Annamaria Massa, Sofia Avnet, Gloria Bonuccelli, Nicola Baldini

**Affiliations:** 1 Orthopaedic Pathophysiology and Regenerative Medicine Unit, Istituto Ortopedico Rizzoli (IOR), 40136, Bologna, Italy; 2 Department of Biomedical and Neuromotor Sciences, University of Bologna, 40126, Bologna, Italy; Second University of Naples, ITALY

## Abstract

Osteosarcoma (OS) is an aggressive bone malignancy with a high relapse rate despite combined treatment with surgery and multiagent chemotherapy. As for other cancers, OS-associated microenvironment may contribute to tumor initiation, growth, and metastasis. We consider mesenchymal stromal cells (MSC) as a relevant cellular component of OS microenvironment, and have previously found that the interaction between MSC and tumor cells is bidirectional: tumor cells can modulate their peripheral environment that in turn becomes more favorable to tumor growth through metabolic reprogramming. Here, we determined the effects of MSC on OS stemness and migration, two major features associated with recurrence and chemoresistance. The presence of stromal cells enhanced the number of floating spheres enriched in cancer stem cells (CSC) of the OS cell population. Furthermore, the co-culturing with MSC stimulated the migratory capacity of OS via TGFβ1 and IL-6 secretion, and the neutralizing antibody anti-IL-6 impaired this effect. Thus, stromal cells in combination with OS spheres exploit a vicious cycle where the presence of CSC stimulates mesenchymal cytokine secretion, which in turn increases stemness, proliferation, migration, and metastatic potential of CSC, also through the increase of expression of adhesion molecules like ICAM-1. Altogether, our data corroborate the concept that a comprehensive knowledge of the interplay between tumor and stroma that also includes the stem-like fraction of tumor cells is needed to develop novel and effective anti-cancer therapies.

## Introduction

The microenvironment of a tumor is heterogeneous. As previously demonstrated both in human carcinomas and sarcomas, a combination of differentiated tumor cells, cancer stem cells (CSC), cancer-associated fibroblasts, mesenchymal stromal cells (MSC), and immune cells form the tumor bulk, and the interaction between these different cell types is required to promote tumor growth and metastasis [1]. Embedded in this complex milieu, CSC are a small subset of tumor cells with stem-like features that are responsible, based on their self-renewing ability and competence to give rise to a differentiated progeny, for tumor initiation and for local and systemic relapse [2]. Given that CSC are the driving force for tumor formation, targeting these cells would hold a substantial potential to improve the outcome of patients treated with conventional anticancer agents. Thus, the successful targeting of this cell population is of utmost importance and represents a critical area of investigation. CSC have been identified in a number of tumors and indeed CSC-like chemoresistant elements have already been identified also in osteosarcoma (OS) [3,4,5,6]. OS is the most common primary malignant bone tumor with a high incidence in childhood and adolescence [7]. Despite the introduction of chemotherapy has raised patient survival from 10% to 65% [8], the clinical outcome has reached a plateau over the last decades [9, 10]. Recurrence usually manifests as pulmonary metastases that occur within 6 months since diagnosis and considerably impact prognosis. Thus, dissecting the mechanisms underlying the development, progression, and metastasis of OS is highly desirable.

According to the leading hypothesis, OS tumor cells originate from MSC, non-hematopoietic precursors residing in the bone marrow, that contribute to the maintenance and regeneration of a variety of tissues, including bone [11]. The existing literature on the pro-tumorigenic vs the anti-tumorigenic effects of MSC is controversial [12]. Despite several studies suggest MSC as an anti-tumor agent [13], their use to counteract cancer growth displays a number of risks. In this view, Perrot *et al*. have reported the case of a local recurrence of OS after an autologous lipofilling procedure and suggested that MSC highly enriched in the fat graft may have promoted tumor growth [14]. Moreover, several reports indicate that MSC produce soluble factors involved in cell proliferation and therefore exert a proliferative and proinflammatory effect on cancer cells [15, 16, 17]. Importantly, it has been shown that MSC can migrate to the OS lesion and contribute to tumor progression in mice [18] and rats [19], therefore cooperating with tumor cells in the development of a suitable microenvironment.

MSC are known to secrete many cytokines; it is widely accepted that cytokine production by tumor-associated stroma can stimulate tumor sustenance, growth, and angiogenesis [20], and that this occurs also in OS [21]. Of these factors and cytokines, interleukin-6 (IL-6) plays a leading role. Originally identified as a T-cell-derived-cytokine that is necessary for the complete transformation of B cells into antibody-secreting plasma cells [22], IL-6 exhibits a plethora of biological activities. Indeed, IL-6 is increasingly recognized as the soluble mediator linking chronic inflammation to cancer development, and its protein and mRNA are often overexpressed in serum and tumor samples from breast, bone, liver and colon cancers both in humans and mice. Moreover, the inhibition of IL-6 signaling is able to slow the growth of colon and lung cancers [23], unveiling a role for IL-6 as a possible therapeutic target.

An increasing number of literature describes the interactions between tumor cells and its niche. We have previously found that the cross-talk between MSC and OS tumor cells is bidirectional, i.e. tumor cells can modulate their peripheral environment that, in turn, becomes more favorable to tumor growth through metabolic reprogramming [24]. Yet, the effects of the microenvironment on OS stem cells have never been explored. Here, we investigated the features of OS CSC grown in the close proximity to MSC and showed that tumor-associated stroma plays a role in supporting stemness, migration and proliferation of the stem-like tumor component. In turn, OS CSC can modulate the healthy surrounding tissue to a pro-tumorigenic behavior by inducing the secretion of pro-inflammatory cytokines, such as Trasforming Growth Factor beta-1 (TFβ1) and IL-6.

## Materials and Methods

### Cell lines and sphere cultures

Unless otherwise stated, all reagents were purchased from Sigma-Aldrich. HOS and MG63 cell lines and bone-marrow MSC were purchased from the American Type Culture Collection (ATCC) and cultured, in IMDM (Life Technologies) for tumor cells or alpha-modified Minimum Essential Medium (αMEM) for MSC plus penicillin (20U/mL), streptomycin (100 mg/mL), and 10% heat-inactivated fetal bovine serum (complete medium). Cells were maintained at 37°C in humidified atmosphere with 5% CO_2._

Sphere-forming cells were obtained as previously described [3]. Briefly, tumor cells were cultured in anchorage-independent conditions in CSC complete medium [DMEM:F12 medium with progesterone (20 nM), putrescine (10 mg/mL), sodium selenite (30 nM), apo-transferrin (100 mg/mL), and insulin (25 mg/mL), human epidermal growth factor (20 ng/mL) and basic fibroblast growth factor (10 ng/mL, PeproTech)], in low-attachment flasks (Nunc). MSC cells that had been cultured for more than 6 passages were never used.

### Co-culture experiments

Before performing all co-cultures, experimental plates were coated with Poly(2-hydroxyethyl methacrylate) (Poly-hema) diluted in 96% ethanol to inhibit cell adhesion; plates were allowed to dry, washed twice in Phosphate Buffer Solution (PBS) and sterilized under UV light.

For co-culture experiments, MSC were seeded in transwell inserts with 0.4 μm pores and allowed to grow in αMEM complete medium for 24 hours. The following day, cells were washed and medium was changed to αMEM plus 0.1% FBS. Cells were maintained in low-serum conditions for the following 24 hours to allow cytokine secretion and accumulation. The following day, osteosarcoma cells were detached, counted and seeded at a density of 10,000 cells/mL in CSC complete medium on Poly-hema coated plates in the lower compartment of a boyden chamber. The pretreated MSC that were previously attached on transwells (or medium only containing transwells) were then added as the upper compartment of the same boyden chamber, and spheres were allowed to grow for 6 days. To assess sphere formation, bright-field pictures of the whole well were taken at 4x magnification. All the spheres in a single well were counted and the diameter of each sphere was measured with ImageJ software. To define a sphere, only cell aggregates with more than 50 μm in diameter (roughly corresponding to spheres of at least 10 cells) were included in the cell counts and in the statistical analysis.

To evaluate sphere growth dependency from IL-6, neutralizing anti-IL-6 monoclonal antibody (Tocilizumab, Roche) was added to MSC medium at the final concentration of 100 μg/mL. Cells were repeatedly exposed to the antibody every 24 hours. For each assays three replicates were performed.

### Proteome profiler array

To assess expression of stem cell-related markers in CSC from osteosarcoma that were co-cultured with MSC and in respect to parental cell line, protein extracts were obtained and quantified with Proteome Profiler Human Pluripotent Stem Cell Array Kit (R&D) lysis buffer. Equal amounts of protein lysates (200 μg) were quantified by Bradford, and then loaded on each membrane. According to manufacturer’s instructions, each sample was loaded as duplicate. The signal of each spot was quantified by dedicated software for densitometric evaluation (VisionWorksLS Analysis Software, Biospectrum, UVP).

### Immunofluorescence

For Ki67 staining, HOS-CSC spheres were trypsinized and counted. Single cells were centrifuged at 8,000 rpm for 8 min with Cyto-Tek centrifuge (Electron Microscopy Science) and, next, fixed in 3.7% paraformaldehyde 20 min. Cells were incubated with monoclonal anti-Ki67 (MIB-1, 1:75, DAKO) for 30 min at 37°C, followed by a secondary anti-mouse FITC-conjugated antibody (1:1000, Chemicon). Nuclei were counterstained with Hoecst 33342. Cells were observed by confocal microscopy (Nikon TI-E). All the cells in a single acquired image were counted and the results were expressed as percentage of positive Ki67 nuclei/total number of cells. The experiment was performed with three replicates.

### Soft-agar assay

Anchorage-independent growth was determined in 0.33% agarose (SeaPlaque; FMC BioProducts) with a 0.5% agarose underlay. After 3 days of co-colture with or without MSC, as previously described, HOS-CSC spheres were trypsinized and counted and plated (1,000 cells/dish) in semisolid medium (IMDM 10% FCS plus agar 0.33%), and incubated at 37°C in a humidified 5% CO_2_ atmosphere. Colonies with a diameter higher than 200 μm were counted after 7 days. The experiment was performed with four replicates.

### TGFβ1 assay

TGFβ1 was measured in the culture media with TGFβ1 Duoset human ELISA kit (R&D Systems) according to the manufacturer’s instructions. The absorbance was read by using Infinite® 200 PRO plate reader (Tecan). The amount of TGFβ1 detected was normalized on the total protein content of the culture media, as evaluated by Bradford method (Bio-Rad). The experiment was performed with six replicates.

### RNA isolation and gene expression

RNA was extracted with NucleoSpin RNA II (Macherey-nagel) and the retrotranscription was performed with MuLV Reverse Transcriptase (Applied Biosystems). Real-Time Polymerization Chain Reaction (Real-time PCR) was performed by amplifying 500 ng of cDNA using the Light Cycler instrument and the Universal Probe Library system (Roche Applied Science). Probes and primers were selected using the web-based assay design software (www.universalprobefinder.com, Roche). For specific primer sequences see Table 1.

**Table 1:** Primers and probes. Gene symbol, name, function, accession number, primer sequences and selected probe are shown.

Gene	Full name	Accession Number	Primers	Probe
**GAPDH**	Glyceraldehyde 3-phosphate dehydrogenase	NM_002046.3	• F = agccacatcgctcagacac • R = gcccaatacgaccaaatcc	60
**TGFβ1**	Trasforming growth factor, beta 1	NM_000660.4	• F = agtggttgagccgtggag; • R = tgcagtgtgttatccctgct	68
**RelA**	v-rel reticuloendotheliosis viral oncogene homolog A (avian)	NM_021975.3	• F = actgtgtgacaaggtgcagaa • R = cacttgtcggtgcacatca	64
**RelB**	v-rel reticuloendotheliosis viral oncogene homolog B	NM_006509.3	• F = gattgtcgagcccgtgac • R = ccacgccgtagctgtcat	4
**Met**	met proto-oncogene (hepatocyte growth factor receptor)	NM_001127500.1NM_000245.2	• F = cagagacttggctgcaagaa • R = ggcaagaccaaaatcagca	42
**ICAM-1**	intercellular adhesion molecule 1	NM_000201.2	• F = ccttcctcaccgtgtactgg • R = agcgtagggtaaggttcttgc	71
**Sox2**	Homo sapiens SRY (sex determining region Y)-box 2	NM_003106.3	• F = gggggaatggaccttgtatag • R = gcaaagctcctaccgtacca	65
**Oct4**	POU class 5 homeobox 1 (POU5F1), transcript variant 1	NM_002701.4	• F = cttcgcaagccctcatttc • R = gagaaggcgaaatccgaag	60
**Nanog**	Nanog homeobox	NM_024865.2	• F = atgcctcacacggagactgt • R = agggctgtcctgaataagca	69
**CXCR4**	chemokine (C-X-C motif) receptor 4, transcript variant 1	NM_001008540.1 NM_003467.2	• F = ggtggtctatgttggcgtct • R = actgacgttggcaaagatga	18
**Galectin-3**	Galectin 3	NC_000014.9	• F = cttctggacagccaagtgc • R = aaaggcaggttataaggcacaa	3
**STAT3**	signal transducer and activator of transcription 3 (acute-phase response factor), transcript variant 1	NM_139276.2	• F = cccttggattgagagtcaaga • R = aagcggctatactgctggtc	14

The protocol of amplification was: 95°C for 10 minutes; 95°C for 10 seconds, 60°C for 30 seconds, and 72°C for 1 second for 45 cycles; 40°C for 30 seconds. GAPDH was used as housekeeping gene to normalize the expression of the genes of interest [25]. The results were expressed as a ratio between gene of interest and GAPDH gene. All experiments were performed with three replicates.

### NF-kB assay

To assess nuclear translocation of NF-κB components RelA and RelB, nuclear protein extracts were obtained and quantified with a Nuclear Extraction Kit (Cayman Chemical). Equal amounts of protein lysates were then used for NF-kB Transcription Factor Assay Kit quantification (TransAM, ActiveMotif) according to manufacturer’s instructions. The signal was quantified by using Infinite® 200 PRO plate reader (Tecan). The experiment was performed with four replicates.

### IL-6 assay

IL-6 was measured in the culture media with IL-6 Duoset human ELISA kit (R&D Systems) according to the manufacturer’s instructions. The absorbance was read by using Infinite® 200 PRO plate reader (Tecan). The amount of IL-6 detected was normalized on the total protein content of the culture media as evaluated by Bradford method (Bio-Rad).

To evaluate IL-6 secretion from MSC in the absence of TGFβ1 during the co-culturing with CSC, a monoclonal anti TGFβ1 neutralizing antibody (1 μg/mL, 1D11, R&D Systems) was added to MSC medium at the same moment in which CSC cells were added to the culture. The TGFβ1 antibody were repeatedly added to the culture every 24 hours. The experiment was performed with six replicates.

### Migration assay

Transwells with uncoated permeable support and 8 μm pores were used. 4x10^4^ viable HOS cells, obtained from trypsinized CSC spheres, were seeded in the top compartment of a boyden chamber, and incubated in 5% CO_2_ at 37°C for 3 hours. The bottom compartment was either left uncoated or seeded with 2x10^4^ MSC; anti-IL-6 monoclonal antibody (100 μg/mL Tocilizumab) was added to MSC medium 2 hours prior CSC plating. After 3 hours, cells attached to the upper surface of the filter were mechanically removed by scrubbing with cotton swabs. Chambers were stained in 0.5% crystal violet diluted in 100% methanol for 30 min, rinsed in water and examined under bright-field microscopy. Values for migration were obtained by counting 5 fields per membrane (20X objective) and represent the average of three independent experiments.

### Western blotting

HOS CSC were harvested in hot lysis buffer (SDS 10% and TRIS HCl 0.5 M pH 6.8) containing protease and phosphatase inhibitors and centrifuged at 13,000x g for 10 minutes at 4°C to remove insoluble debris. Protein concentrations were analysed using BCA Protein Assay Kit (Thermo Fisher Scientific). 30 μg of proteins were loaded and separated by SDS-PAGE and transferred to a 0.2 μm nitrocellulose membrane (Thermo Fisher Scientific). After blocking for 1 hour in TBST (10 mM Tris-HCl pH 8.0, 150 mM NaCl, 0.5% Tween-20) with 5% non-fat dry milk, membranes were incubated with the primary antibody for 1 hour or overnight, washed and incubated for 1 hour with horseradish peroxidase-conjugated secondary antibodies (Amersham). The membranes were washed and incubated with an enhanced chemi-luminescence substrate (ECL; Thermo Fisher Scientific). The antibodies were as follow: polyclonal anti-GAPDH (1:1000; Santa Cruz); monoclonal ICAM-1 (mem-111, 1:1000; Abcam). The signal from each band was quantified by dedicated software for densitometric evaluation (VisionWorksLS Analysis Software, Biospectrum, UVP). We performed three replicates.

### CXCR4 assay

Chemokine (C-X-C motif) receptor 4 (CXCR4) was measured in cell lysates with Human CXCR4 ELISA kit (LSBio) according to the manufacturer’s instructions. The signal was quantified by using Infinite® 200 PRO plate reader (Tecan). Cells were lysed by freeze and thaw in PBS, and quantified by Bradford reagent. The amount of CXCR4 detected was normalized on the total protein content of the cell lysates, as evaluated by Bradford method (Bio-Rad). The experiment was performed with four replicates.

### Statistical analysis

Quantitative results were expressed as arithmetic mean plus or minus the standard error of the mean (SEM), from at least three independent experiments. Data were considered as non parametric. Mann-Whitney test was performed for unpaired comparison of two independent variables. Wilcoxon signed rank test was performed for the statistical paired analysis of stem-related markers between the two groups (CSC co-cultured with MSC vs CSC cultured alone). All p values <0.05 were considered as statistically significant. The statistical analysis was performed by StatView5.01 software (SAS Institute Inc.).

## Results

### Stromal cells increase the number, stemness and proliferation of HOS-CSC spheres

**Fig 1:**
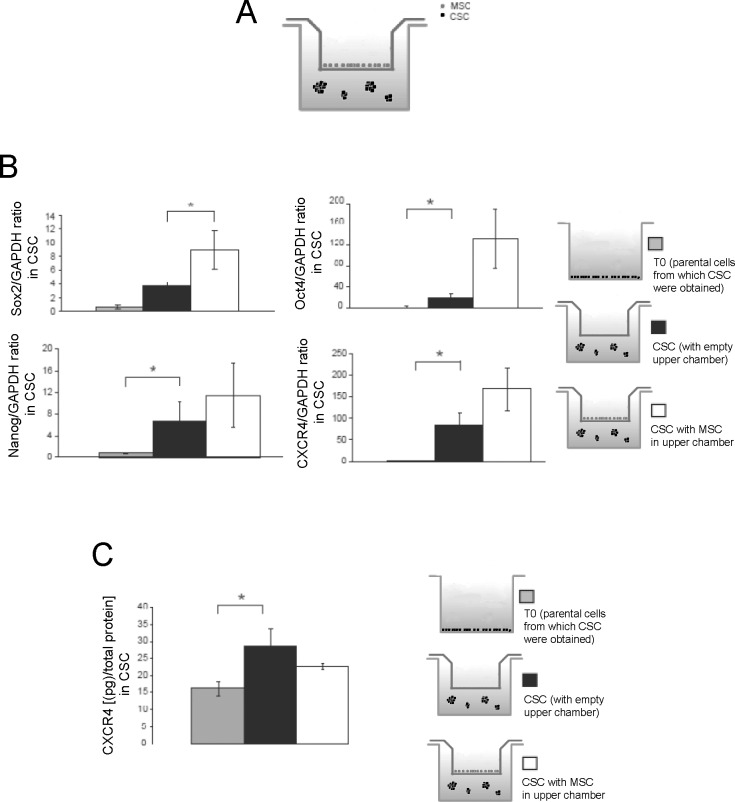
Gene expression of stem-cell markers confirmed the enhancement of stem-like features of CSC when are co-cultured with MSC in transwell. **(A)** Scheme of the co-culture system of HOS-CSCwith MSC used in this study. The co-culturing was prolonged for 3 days; **(B)** Sox2, Oct4, Nanog, and CXCR4 expression evaluated by Real Time PCR in HOS-CSC spheres that were cultured alone or with MSC (in transwell). Gene expression of CSC was also compared to parental HOS adherent cells (T0) (*p<0.05); **(C)** CXCR4 was also evaluated by ELISA. (*p<0.05). Note that the levels of Oct4, Nanog, and CXCR4 markers in the spheres were significantly higher than the parental cell line. For Sox2, we saw a similar trend of increase in CSC spheres respect to parental cells, although this increase was more evident when CSC where co-cultured with MSC.

To obtain HOS-derived CSCs, cells were detached and cultured in serum-free medium and in anchorage-independent conditions. To assess whether the HOS-derived spheres had stem-like features, the mRNA of a number of well-established stem cell genes were analyzed with respect to the parental, adherent HOS cell line, as soon as the sphere were formed (after around 3 days of culture). Indeed, Nanog, Oct4, and CXCR4 were several-fold upregulated in the transition between the adherent cell monolayer (indicated as T0), whereas Sox2 showed only a trend of increase (Fig 1).

CXCR4 increased expression was confirmed also at protein level (Fig 1).

**Fig 2:**
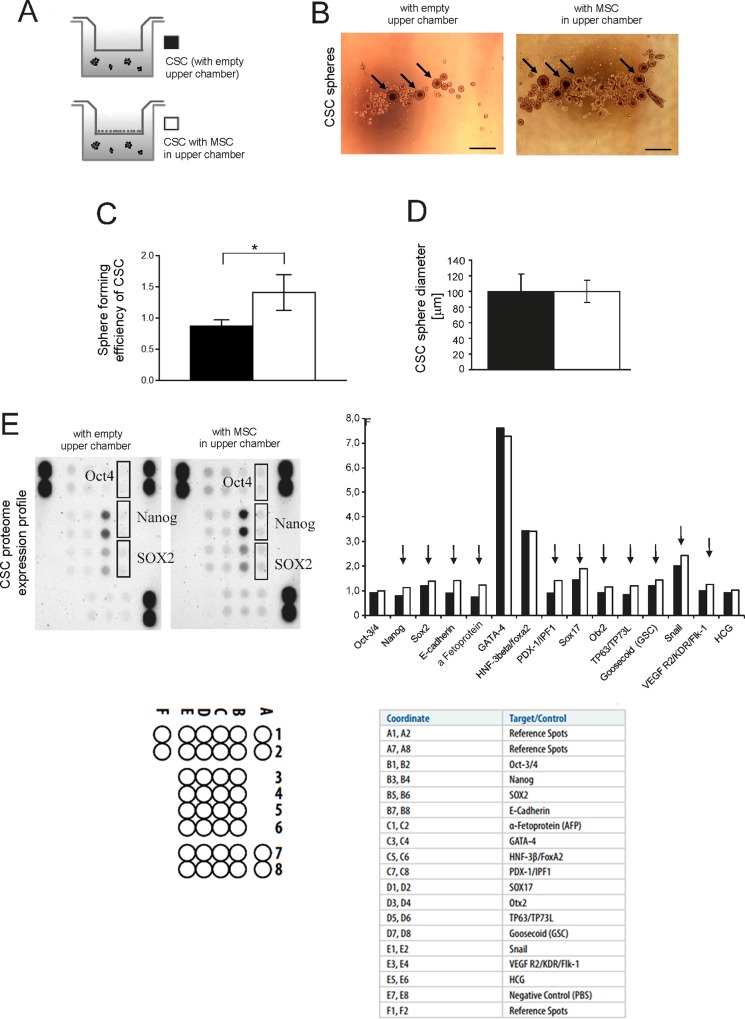
The co-culturing of OS cells with MSC enhances the spherogenic potential and protein expression of stem-related markers in OS-CSC spehers. **(A)** Scheme of the assays showed in this figure. **(B)** Representative pictures of the formed sphere of CSC in different conditions: with or w/o MSC in the upper chamber, see panel A (scale bar 500 μm, black arrows show the spheres with higher size); **(C)** spheres of CSC shown in panel A were counted and expressed as sphere forming efficiency (number of spheres formed / number of cells seeded × 100) (*p<0.05); **(D)** Average diameter of the counted spheres of CSC in panel C; **(E)** Stem cell markers of CSC spheres were evaluated by proteome expression profiler (left panel, image of the blotted membranes; right panel, densitometric evaluation of the same membranes and graphical representation of the obtained data); Oct4, Sox2 and Nanog have been highlighted on the membrane. Map of the blotted membrane and human pluripotent stem cell array coordinates are also shown.

We then co-cultured CSC with bone marrow-derived MSC (hereon only MSC) that were plated in the upper compartment of a Boyden chamber. After 6 days of culture, we observed a significant increase in sphere number (Fig 2, panels A and B) but no difference in sphere diameter (Fig 2C).

**Fig 3:**
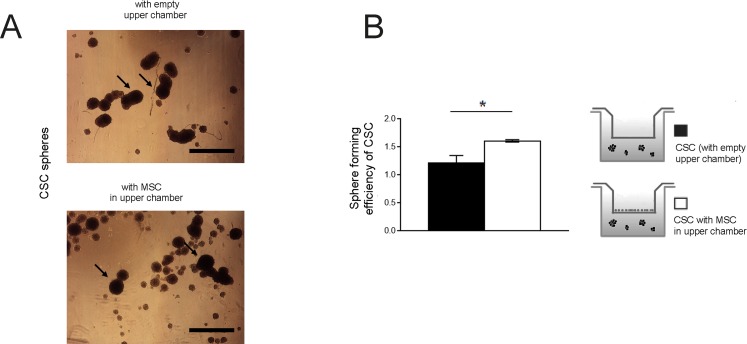
MG63 OS cells form spheres and their number is increased by the presence of MSC. **(A)** Homotypic cultures of CSC and their co-culture with MSC for 6 days were photographed at a magnification of 4x, representative images (scale bar 500 μm, black arrows show the spheres with higher size); **(B)** spheres were counted and expressed as sphere forming efficiency (number of spheres formed / number of cells seeded × 100) (*p<0.05).

Furthermore, we observed a trend of increased expression for Nanog and Oct4, and a significant increased expression for SOX2 (Fig 1). The same proved to be true for another OS cell line, namely MG63 (Fig 3).

To confirm stemness of the observed spheres in HOS cells formed when co-cultured with MSC, stem markers were evaluated by proteome profiler array. Protein analysis showed for a number of stem cell-related markers, including Oct-4, Nanog, and SOX2, a trend of up-regulation in HOS-CSC co-cultured in the presence of MSC, respect to CSC alone (Fig 2D and 2E). Notably, the statistical analysis of the two paired groups (CSC co-cultured with MSC vs CSC cultured alone) revealed that the increased expression of the stem-related markers at protein level was significant (*p < 0.05).

**Fig 4:**
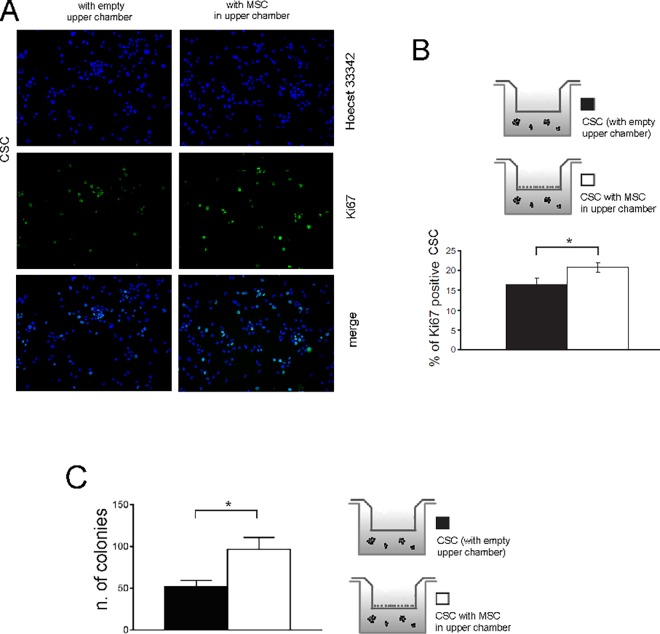
MSC increase the proliferation of HOS-CSC. Homotypic cultures of CSC or co-cultures of CSC+MSC for 3 days were trypsinized, fixed and immunostained with anti-Ki67 antibody (green). Hoecst 33342(blue) was used to counterstain nuclei. **(A)** Representative image; **(B)** The percentage of Ki67 positive nuclei was quantified and expressed as ratio to the total number of cells (*p<0.05). Ki67 expression was increased in the presence of MSC as compared to CSC cultured alone. Images were acquired using the same exposure setting. Original magnification 20X. Figure shows representative images. **(C)** The number of colonies formed by HOS-CSC co-cultured with MSC were evaluated on soft-agar and quantified. MSC secreatome enhanced HOC-CSC tumorigenicity.

Next, we hypothesized that the increased number of HOS-CSC spheres observed in Fig 1A could be due to a higher cell proliferation rate. To test this hypothesis, we performed immunostaining analysis for Ki67 and soft agar assay on HOS-CSC. We found that exposure to MSC increased the number of Ki67 positive cells to a significant extent (Fig 4F), indicating a higher rate of proliferation.

Overall, these data suggest that the presence of the stromal component induces an increase in the stemness features and proliferation of CSC.

### OS stem cells promote the activation of pro-inflammatory pathways in MSC

**Fig 5:**
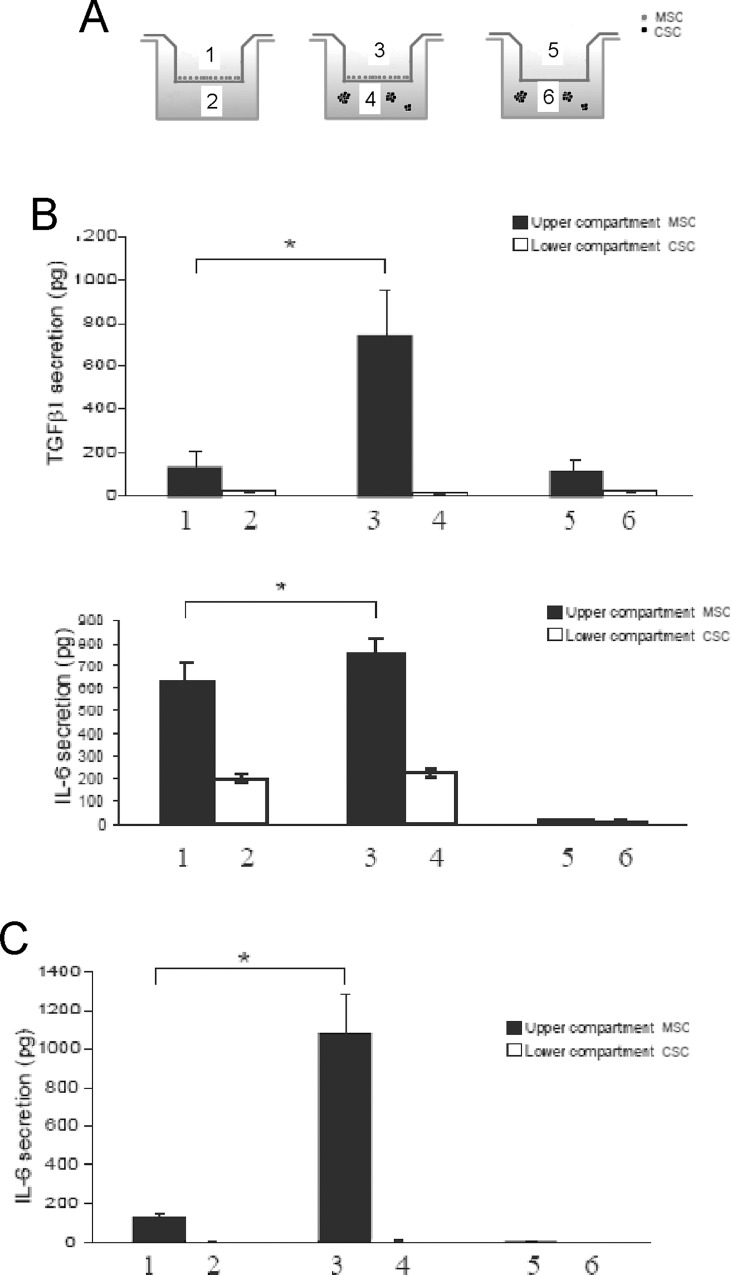
MSC secrete pro-inflammatory cytokines when exposed to OS CSC whereas CSC are unaffected. **(A)** Scheme of the experiments used to evaluate the secreted proteins showed in the following panels; **(B)** TGF- β1 and IL-6 ELISA assays of the supernatants collected from the different chambers of the transwell with MSC co-cultured with HOS-CSC. TGFβ1 is secreted exclusively by MSC co-coltured in the presence of stem-like cells whereaseIL-6 is secreated by MSC independently from the co-cultuing with MSC. However secreated IL-6 levels were increased in MSC when co-cultured with CSC. The amount of IL-6 detected in lanes 2 and 4 is most likely due to leakage from the upper chamber, as no amount could be detected in the control in lane 6, where no MSC were seeded; **(C)** IL-6 ELISA assay for the evaluation of the same phenomenon with MG63-CSC secretion. The trend ofMG63-CSC was the same observed for HOS-CSC cells.

TGFβ1 is one of the most abundant cytokines in the tumor microenvironment. Therefore, we sought to investigate whether the stem-like component of OS could be stimulated at increasing TGFβ1 expression by MSC. We used**B**oyden chambers in which we co-cultured MSC and osteosarcoma CSC for 3 days according to the scheme showed in Fig 5A. Interestingly, we observed a dramatic increase in secretion exclusively in MSC compartment (upper compartment), rather than in CSC compartment (lower compartment) (Fig 5).

**Fig 6:**
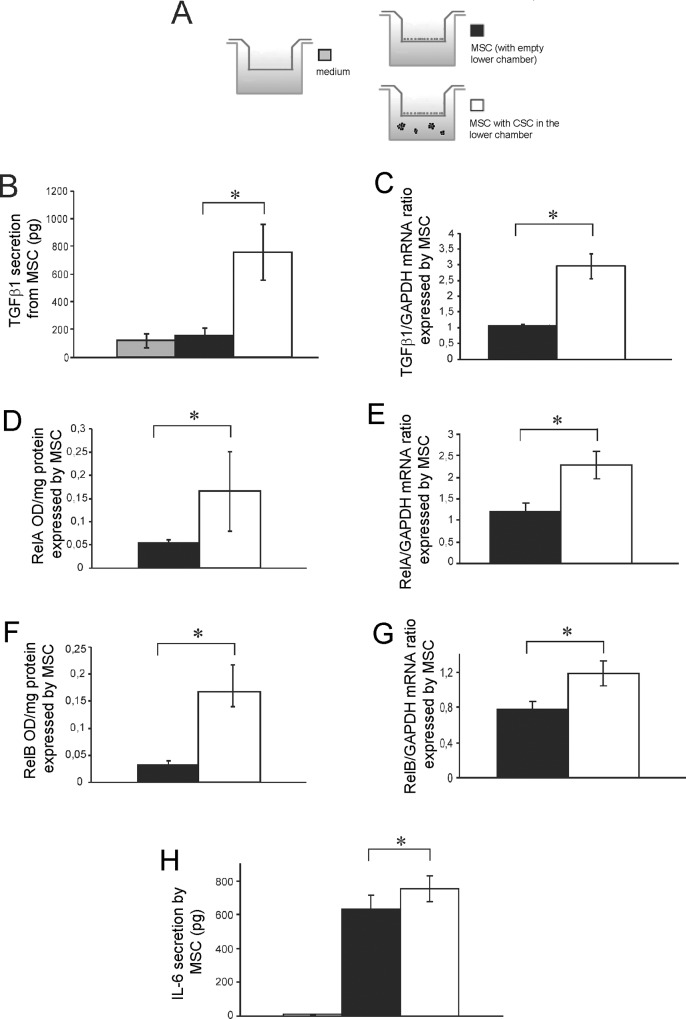
The presence of HOS-CSC enhance the secretion of TGFβ1 from MSC that, in turn. autocrinally induce the activation of inflammatory pathways. **(A)** Scheme of the experiments used to evaluate the secreted proteins showed in the following panels. The secreted protein (only the supernatant in the MSC upper compartment was) **(B)** and mRNA expression **(C)** of TGFβ1 in MSC when co-cultured with HOS-CSC, after 3 days (*p<0.05). The presence of HOS-CSC, increased the expression levels in MSC of two genes of the NF-kB pathway, RelA **(D** for protein and **E** for mRNA**)** and RelB **(F** for protein and **G** for mRNA**)**, as shown by direct NF-kB nuclear translocation (D and F panels) or transcriptional activation (E and G panels), *p<0.05. **(H)** When activated by the presence of HOS-CSC, MSC secreted higher amounts of IL-6, as assessed by ELISA assay, (*p<0.05).

In more details, we saw a significant increase of mRNA expression and protein secretion of TGFβ1 in MSC when co-cultured with CSC, in respect to MSC alone (MSC) o in respect to MSC medium (negative control), (Fig 6B).

We next set out to assess the activation of pro-inflammatory genes in MSC in the same conditions. Firstly, we evaluated the presence of the nuclear factor kappa-light-chain-enhancer of activated B cells (NF-kB) complex. The NF-kB proteins comprise a family of transcription factors that control cytokine secretion and cell survival. Interestingly, 6 hours of co-culture proved sufficient to profoundly upregulate RelA and RelB when MSC were in proximity to HOS-CSC both at mRNA and protein level (Fig 6D–6G). Since many of the immune response and acute-phase response genes contain a NF-kB binding site, including the gene of IL-6 cytokine, we sought to investigate the CSC-dependent IL-6 secretion in MSC. Indeed, the ELISA results showed that, although MSC secreted already a considerable amount of the pro-tumorigenic cytokine at the basal level, when exposed to the presence of HOS-CSC or to CSC derived from MG63, the levels of IL-6 significantly increased (Fig 6H and Fig 5B for results with HOS-CSC and Fig 5C for results with MG63-CSC). Interestingly, the tumor OS cells showed unambiguously no secretion (Fig 5), supporting the concept that inflammation occurs at the interface between tumor and stroma and is guaranteed by the cancer-activated stromal cells rather than by OS cells.

Overall, the data in Fig 6 suggest that a higher amount of mesenchymal-secreted TGFβ1 could act autocrinally on stromal cells, trigger inflammatory signaling pathways that might activate NF-kB nuclear translocation, and ultimately prompt IL-6 secretion.

### TGFβ1-dependent IL-6 secretion is responsible for OS spheroid formation

**Fig 7:**
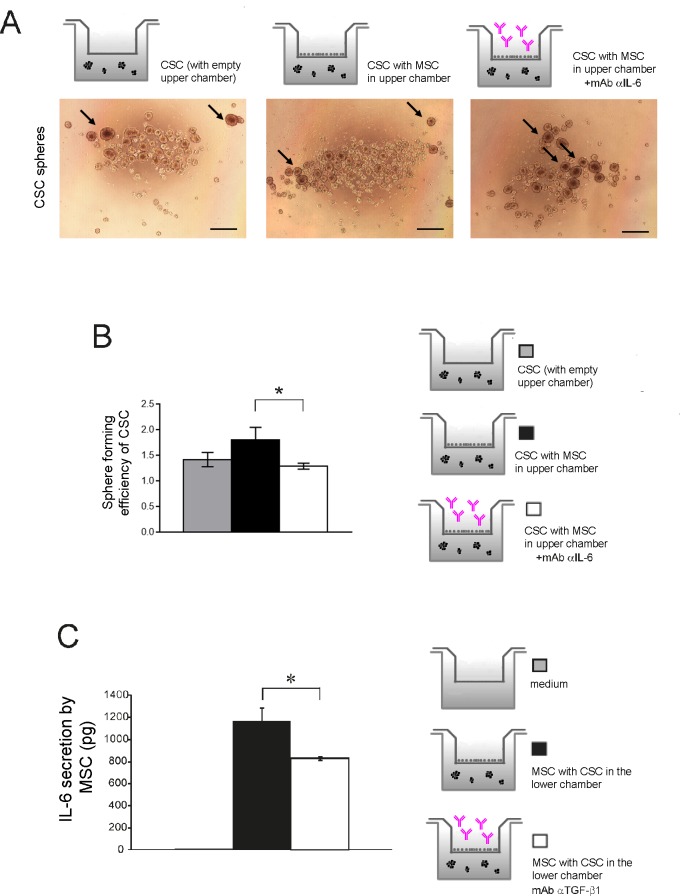
TGFβ1-dependent IL-6 secretion is responsible for increased CSC sphere formation. **(A)** HOS-CSC were let to grow for 6 days in the presence of MSC; the latter cells were added every 24 hours with 100 μg/mL of Tocilizumab. Spheres were then photographed, counted, and quantified (scale bar, 500 μm). Representative figures and schematic representation of the performed assay; **(B)** Treatment with Tocilizumab significantly decreased the number of HOS-CSC spheres showed in panel A (*p<0.05); **(C)** IL-6 secretion by MSC is TGFβ1 dependent. MSC were treated with 1 μg/mL mAb αTGFβ1 2 hours prior CSC seeding, and re-added every 24 hours for the three days of co-culture. Supernatants from the MSC upper compartment were then collected and analyzed for IL-6 secretion by ELISA assay (*p<0.05).

To assess the functional role of IL-6, we exposed MSC to the presence of a mAb that blocks the IL-6 receptor/ligand interaction and evaluated HOS-CSC spheroid formation. Indeed, exposure of MSC to the anti-IL-6 antibody significantly blunted the number of HOS-derived stem-like spheroids, as shown in Fig 7A and quantified in Fig 7B.

Then, we wanted to confirm our hypothesis that MSC-secreted TGFβ1 acts autocrinally on the stromal component and influences IL-6 secretion. We blocked TGFβ1 in the MSC compartment with a specific neutralizing antibody, and measured the amount of secreted IL-6. Indeed, as shown in Fig 7C, IL-6 secretion was significantly reduced.

### MSC increase the expression of adhesion molecules responsible for tumor cell extravasation in an IL-6-dependent fashion

**Fig 8:**
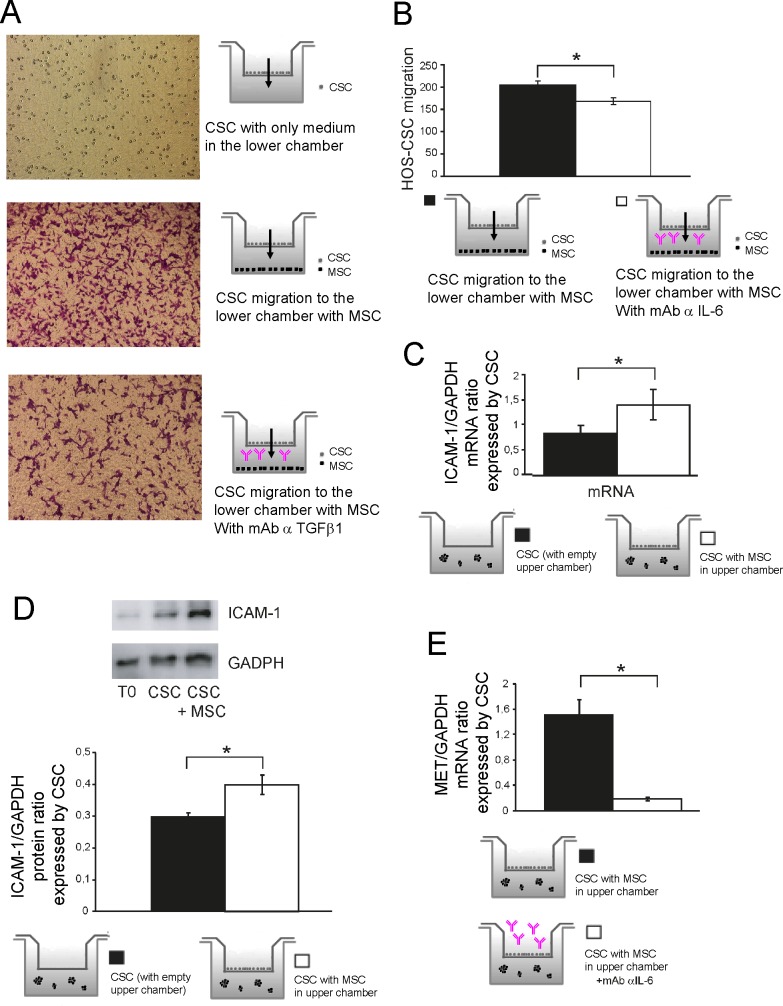
Stromal cells enhance HOS-CSC migration via IL-6 and the expression of adhesion molecules. **(A)** We assessed whether treatment with Tocilizumab affected HOS-CSC migration. MSC were treated with Tocilizumab [100 μg/mL] 2 hours prior CSC seeding. HOS spheres were trypsinized and single cells were let to migrate for 3 hours. As a control, medium only was added in the lower chambers, representative images; **(B)** Quantification of the migration assay shown in panel (A) (*p<0.05); **(C)** The expression levels of ICAM-1 were increased in HOS-CSC co-cultured with MSC. Data were obtained by Real Time PCR (*p<0.05) and confirmed by Western blot **(D,** representative image and densitometric quantification, T0 represents the protein expression level of parental cells from which CSC was obtained) (*p<0.05); **(E)** MSC were treated with 100 μg/mL Tocilizumab 24 hours prior CSC seeding. HOS-CSC spheres were then co-cultured by using tranwells with MSC and incubated for 6 hours. The RNA from CSC was then extracted and analyzed for the MET expression that shows a dramatic decrease in the absence of IL-6 (*p<0.05).

We then further substantiated the role of IL-6 in the context of *in vitro* cell migration. Crystal violet staining of dismembered HOS-CSC that were allowed to migrate in Boyden chambers for three hours showed that MSC pre-treatment with anti-IL-6 antibody was sufficient to significantly reduce the migration potential of OS cells, as shown in Fig 8A and quantified in Fig 8B. These data show that exogenous IL-6 is responsible for the aggressive migratory phenotype of OS stem-like spheroids.

**Fig 9:**
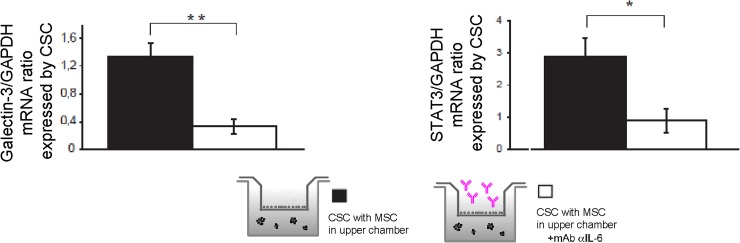
Expression of genes involved in migration confirms a role for IL-6 in CSC metastatic process. Galectin-3 (**p<0.01) and STAT3 (*p<0.05) were evaluated in HOS-CSC spheres after 3 days of co-culturing with MSC, and exposed to mAb anti IL-6 by Real Time PCR.

For cancer cells to metastasize, they must first invade the tissues surrounding the primary tumor; a number of prometastatic genes, including adhesion molecules, transcription factors or cellular receptors, are involved in the process. Intercellular adhesion molecule-1 (ICAM-1) is an inducible surface glycoprotein that mediates adhesion-dependent cell-to-cell interactions, and can also facilitate cell mobility through the extracellular matrix [26]. We therefore examined whether ICAM-1 was overexpressed in the stem-like component of tumor cells. Results showed that stromal cells increased mRNA expression of the adhesion molecule (Fig 8C transcript and 8D protein expression). To validate once more the role of IL-6, CSC were exposed to IL-6-neutralized MSC and transcripts involved in migration were analyzed. ICAM-1 expression proved not to change in response to variations to IL-6 levels (data not shown); nevertheless, MET expression was greatly affected by the anti IL-6 antibody (Fig 8E), suggesting that OS CSC might be stimulated in migration, via IL-6, throughout signaling of the hepatocyte growth factor receptor. MET is an oncogene that, when activated by its ligand HGF, exhibits potent migration/invasion-inducing activity and is frequently expressed in OS and is strictly related to its aggressiveness [27], and found relevant for the pathogenesis of this tumor [28, 29]. Interestingly, also other genes involved in migration, such as galectin-3 and STAT3, were impaired by the absence of IL-6 (Fig 9).

In all, we show that the vicinity to stromal cells increases the expression of adhesion molecules and that IL-6 contributes to their overexpression.

## Discussion

Reactive stromal cells are abundantly present in solid tumors, and their role in tumor progression has been unequivocally demonstrated. In breast and pancreatic cancer, cancer-associated fibroblasts (CAF) play fundamental roles in tumor progression, initiation, and metastasis [30]. By themselves, stromal cells are not malignant and maintain normal tissue structure and function. However, through intercellular interactions or by paracrine secretions by cancer cells, normal stromal cells may acquire abnormal phenotypes that sustain cancer cell growth and tumor progression [31]. The interactions between these diverse components have become increasingly evident and are crucial for tumor development and progression. Since cancer treatment failure is essentially due to the presence of a resistant population of cells, the CSC, in this study, we decided to focus on two OS subpopulations: CSC-enriched cultures and the normal, healthy tissue found in close proximity to cancer cells representing the peripheral environment, i.e. the MSC.

**Fig 10:**
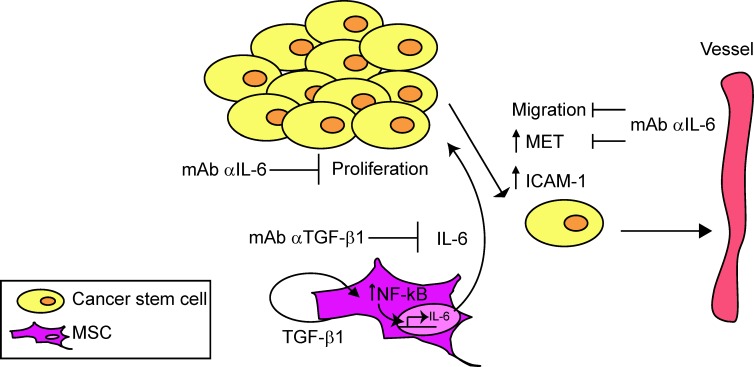
Model for the circuit between MSC and HOS-CSC. Recruitment of MSC to the tumor environment leads to enhanced proliferation of OS stem cells. The presence of CSC, in turn, leads to a consistent secretion of TGFβ1 that activates a stromal autocrine loop that might be responsible for the activation of NF-kB genes and IL-6 secretion by MSC. Indeed, neutralization of TGFβ1 reduces the amount of secreted IL-6. Pro-tumorigenic effects of MSC, via IL-6, including induction of HOS-CSC migration and sphere growth, can be counteracted also by IL-6 neutralizing antibody. The presence of MSC is also responsible for increased expression of adhesion molecules involved in intra- or extra-vasation and the expression of MET can be counteracted by IL-6 neutralization.

Our results suggest that the interaction between the tumor stem-like component and the reactive stroma generates a vicious circle that is capable of self-sustenance by which MSC, after having been in contact with OS cells, increase the aggressive behavior of cancer cells (Fig 10).

We propose a model of recruitment of MSC to the tumor environment that, in turn, leads to stimulation of CSC growth. In particular, we showed an increase in floating sphere number and a low but consistent increase in the percentage of Ki67-positive nuclei. As proof-of-principle of the enrichment of CSC-like cells in the low-adherence HOS culture, we analyzed a panel of widely-accepted stem-cell genes; we demonstrated the higher level of expression of the stemness related factors Oct4, Nanog, CXCR4, and Sox2 as compared to the native, adherent culture. Moreover, we could demonstrate that the presence of the stromal compartment enhances the stemness properties of HOS-CSC by increasing, significantly, the expression of Sox2 and leading to a trend of increase for a plethora of stem cell markers. Importantly, the proteome profiler array revealed that the increased protein expression of the stem-related markers here considered was significant, when compared between the two paired groups (CSC co-cultured with MSC vs CSC cultured alone). Interestingly, we analyzed for the first time the expression of MET in the stem-like component of OS and demonstrated a significant increase when spheroids were co-cultured along with stromal cells.

We also found evidence for the activation of the inflammatory pathways mediated by NF-kB in the MSC compartment. The role of NF-kB in cancer is manifold and complex [32, 33]; NF-kB induces the secretion of pro-tumorigenic cytokines, such as IL-6, and this, in turn, is likely to have a paracrine effect on OS cells via activation of the STAT3 pathway [34, 35], a well known activator of cell proliferation [36], suggesting that stromal cells co-evolve with tumor cells to enhance tumor aggressiveness. In cancer, IL-6 activates target genes involved in a number of biological activities such as differentiation, survival, apoptosis, and proliferation and also modulates tumor cell migration and progression [37, 38]. Furthermore, it is known that OS behaviour is adversely influenced by osteoclast bone-resorption and by IL-6 levels at the tumor site [39] and that IL-6 can increase chemoresistance of human OS cells [40]. However, the role of IL-6 on human OS stem cells is yet unexplored. Using a monoclonal neutralizing antibody, we found that IL-6 is responsible for CSC sphere growth, suggesting that it could be involved in the maintenance of the population putatively involved in OS relapse. Besides, IL-6 has been reported to stimulate directional migration and invasion of human cancer cells [41, 42]. Indeed, we demonstrated that this is true also for OS CSC, as the migration ability of the cells in the presence of anti IL-6 antibody was deeply impaired. Taken together, our data suggest that IL-6 secreted in tumor environment is associated with the metastatic potential of human OS.

TGFβ1 is one of the most important cytokines secreted by OS cells [43]. Consistently, TGFβ1 has also emerged as a key player in the maintenance of self-renewal and stemness [44]; in particular, Tu *et al*. have demonstrated that the tumor maintains the stemness of MSC through the TGF/Smad3 pathway and that OS cells, via TGFβ1 secretion, enhance the production of pro-tumorigenic cytokines, such as IL-6, in the nearby stroma [34]. In partial discordance with these data, we have demonstrated that OS CSC do not secrete TGFβ1 that is rather secreted by the mesenchymal counterpart and considerably increases when MSC are adjacent to the tumor component. Recent works have uncovered a mechanism by which, in cancer cells, TGFβ1 signals through NF-kB activation and nuclear translocation, ultimately leading to a rise in IL-6 secretion [45, 46, 47, 34]. Our results argue for the first time that TGFβ1 is responsible for MSC self-sustenance via the activation of an autocrine loop and that its neutralization profoundly affects IL-6 secretion, inevitably leading to OS reduction of growth and metastatic potential. It is important to note that OS patients with higher levels of TGFβ1 in respect to healthy controls correlate with the presence of distant metastases [48], suggesting that the secretion of this cytokine might modulate the surrounding stroma to support a more aggressive phenotype in OS cells also *in vivo*.

Tumor cells not only take advantage of the trophic factors released by nearby cells, but also use adhesion molecules, chemokines and receptors to aid in migration and homing to distant sites [49]. Among adhesion molecules, ICAM-1 is upregulated in response to a number of stimuli, and several lines of evidence have shown that, in addition to its role in leukocyte adhesion and cancer cell invasion [50, 51], ICAM-1 also plays a role in IL-6-mediated OS cell motility [52]. We show that ICAM-1 is upregulated in OS CSC exposed to non-tumor component, but could not confirm previous data showing its dependency on IL-6. Despite this, we could observe IL-6-dependent downregulation of a number of genes involved in migration. The hepatocyte growth factor and its receptor have been described to be associated not only with high tumor grade and poor prognosis of a number of cancers, but also with migration and development of distant metastasis [53, 54]. Moreover, MET and IL-.6 signaling have been reported to upregulate each other’s receptors and cooperatively enhance tissue invasion [55, 56]. MET encodes for the tyrosine kinase of the hepatocyte growth factor receptor; stable overexpression of MET results in the conversion of primary human osteoblasts into OS cells, demonstrating that MET is pro-tumorigenic and necessary for the induction of a cancer phenotype [28]. Moreover, MET has been previously reported to correlate with aggressiveness of tumor cells, including OS and rabdomyosarcoma [27, 28, 57, 58, 59]. Indeed, stromal IL-6 neutralization dropped the expression of MET in CSC. MET shows therefore an increasingly important role in osteosarcoma, as its overexpression seems to be linked to migration and invasion of CSC. An IL-6-dependent downregulation of migration genes was observed also for STAT3 and galectin-3. The former is an upstream target of the IL-6 signaling pathway; its downregulation proves efficient IL-6 inhibition and possibility contributes to a blockade in cell proliferation [60]. The latter promotes β-catenin/Wnt signaling, and β-catenin-related oncogenesis has been frequently reported in osteosarcoma; its silencing is associated with inhibited secretion of IL-6 [61]. Overall, we show that the vicinity to stromal cells increases the expression of adhesion molecules and that IL-6 contributes to their overexpression.

## Conclusions

Here, for the first time, our observations provide evidence that MSC can act as modulators of OS CSC aggressiveness and metastatic potential. Together with recent reports showing that IL-6 suppression reduces OS seeding and metastasis [62], our study paves the way to the hypothesis that anti IL-6 therapies might be used in combination to standard chemotherapy in the treatment of OS to reduce metastatic spread. Of great importance, for the first time in OS, we show that elevated levels of IL-6 and TGFβ1 are provided by stromal cells, rather than tumor cells and highlight the importance of taking into careful consideration the non-tumor component of the malignancy. In conclusion, OS CSC represent an attractive culprit for the relapse rate that plague OS patients and our data suggest that strategies designed to specifically target the dangerous liason between CSC and its microenvironment represent an important approach to improve patient outcome [63].
